# Rapid evolutionary adaptation to growth on an ‘unfamiliar’ carbon source

**DOI:** 10.1186/s12864-016-3010-x

**Published:** 2016-08-24

**Authors:** Zvi Tamari, Avihu H. Yona, Yitzhak Pilpel, Naama Barkai

**Affiliations:** Department of molecular genetics, Weizmann institute of science, Rehovot, 76100 Israel

**Keywords:** Evolution, Xylulose, Amino-acid biosynthesis

## Abstract

**Background:**

Cells constantly adapt to changes in their environment. When environment shifts between conditions that were previously encountered during the course of evolution, evolutionary-programmed responses are possible. Cells, however, may also encounter a new environment to which a novel response is required. To characterize the first steps in adaptation to a novel condition, we studied budding yeast growth on xylulose, a sugar that is very rarely found in the wild.

**Results:**

We previously reported that growth on xylulose induces the expression of amino acid biosynthesis genes in multiple natural yeast isolates. This induction occurs despite the presence of amino acids in the growth medium and is a unique response to xylulose, not triggered by naturally available carbon sources. Propagating these strains for ~300 generations on xylulose significantly improved their growth rate. Notably, the most significant change in gene expression was the loss of amino acid biosynthesis gene induction. Furthermore, the reduction in amino-acid biosynthesis gene expression on xylulose was tightly correlated with the improvement in growth rate, suggesting that internal depletion of amino-acids presented a major bottleneck limiting growth in xylulose.

**Conclusions:**

We discuss the possible implications of our results for explaining how cells maintain the balance between supply and demand of amino acids during growth in evolutionary ‘familiar’ vs. ‘novel’ conditions.

**Electronic supplementary material:**

The online version of this article (doi:10.1186/s12864-016-3010-x) contains supplementary material, which is available to authorized users.

## Background

Growing cells need to adjust the rate of biomass accumulation with the cell division time. Tight regulation of protein synthesis rate is particularly critical, as it consumes a large fraction of cellular resources. Indeed, studies over the past decades have shown that expression of translation-related genes, such as those required for the production of ribosomes, are modulated in proportion to cellular growth-rate in rapidly growing cells such as microorganisms or cancer cells [1, 2]. In the budding yeast, for example, expression of genes coding for ribosomal proteins (RP), proteins required for ribosome biogenesis (Ribi) or other translation related factors, strongly correlated with cellular growth rate in various environments, and can in fact be used to predict growth rate [3–7].

Amino acids provide the substrate for protein synthesis and their influx should similarly be coordinated with growth rate. Amino acids are taken up from the environment using specialized transporters. In addition, most amino-acids can be produced *de-novo* as part of cellular metabolism. Pathways involved in *de-novo* synthesis are induced at the transcriptional level when intra-cellular amino acids are depleted [8–11]. In particular, depletion of amino acids leads to the accumulation of uncharged tRNAs, leading to increased translation of GCN4, the major transcription factor inducing the expression of dozens of genes required for amino acid biosynthesis [12–16]. This pathway is known to be readily induced under conditions of amino acid limitation. However, expression of this pathway is not modulated in response to environmental changes as ribosome-associated genes are, and does not appear to correlate with growth rate. Specifically, shifting cells between different carbon sources rapidly modulated the expression of RP and Ribi genes, but did not significantly alter the expression of genes required for amino acid biosynthesis. This indicates that the capacity of translation machinery (ribosomes), rather than amino acid de-novo production, is adjusted according to the metabolic capacity upon varying conditions, thereby ensuring optimal protein production.

We recently reported a notable exception to this behavior while studying the transcription profile of different budding yeast strains upon shift from glucose to xylulose, the only pentose sugar budding yeast are able to metabolize [17]. Xylulose is phosphorylated to xylulose-5-phosphate, an intermediate metabolite of the pentose phosphate pathway and thus a substrate for cellular metabolism. However, xylulose is not naturally available in significant amounts outside of living cells [18, 19] and can therefore be viewed as an ‘unfamiliar’ or ‘novel’ carbon source for which yeast did not undergo evolutionary adaptation. Inter-strain diversity in growth rate and ethanol production was significantly higher during growth on xylulose than that observed during growth on other ‘familiar’ carbon sources such as glucose and galactose. While this large diversity could be a sign of local adaptation [20], it could also reflect the rarity by which this carbon source is encountered in nature.

Measuring expression profiles of twenty four budding yeast strains growing on xylulose we noted that no correlation was observed between growth rate and expression of the gene groups that were shown to be tightly correlated with growth rate during growth on different carbon sources (e.g. RP and Ribi). Instead, xylulose strongly induced expression of amino acid biosynthesis genes. Furthermore, the level of induction was significantly (anti)correlated with growth rate on xylulose. Searching for additional conditions that activate this gene group in a growth rate-correlated manner, we identified a large number of gene deletion mutants which may similarly represent conditions not optimized by evolution [21]. Based on this observation, we hypothesized that familiarizing cells to such ‘novel’ conditions by evolutionary adaptation might restore the typical expression profile.

To test this prediction, we evolved twelve wild-type budding yeast strains on xylulose for ~300 generations and compared their gene expression profiles before and after this evolution process. As predicted, we found that induction of amino acid biosynthesis genes was practically eliminated in the evolutionarily-optimized cells. In addition, the degree of growth improvement was correlated with the change in expression of this gene group. We discuss the implications of our results for explaining how cells maintain the balance between amino acid supply and demand for optimizing protein synthesis and growth rate in ‘familiar’ vs. ‘novel’ conditions.

## Results

### Microevolution improved growth on xylulose of multiple strains

**Fig. 1:**
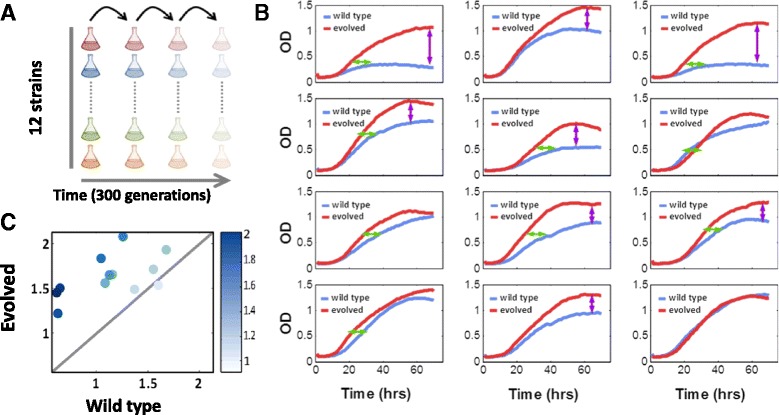
Evolutionary adaptation to growth on xylulose. **a** Twelve diverse wild type yeast strains were grown in parallel on xylulose as the carbon source and propagated for ~300 generations by serial dilution. **b** Growth curve (OD_600nm_) of each strain grown on xylulose, before (*blue*) and after (*red*) evolution. Arrows indicate the evolutionary effect on cell density (*purple*) and growth rate (*green*). **c** Effective growth rate of wild type vs. evolved strains. Color code represents the level of improvement in effective growth rate during evolution defined as the ratio between respective effective growth rates

We previously reported the growth rate and gene expression of a collection of twelve wild type *S. cerevisiae* strains, isolated from diverse geographical origins and natural habitats, on multiple carbon sources including xylulose [17]. All of these strains could grow on xylulose, although their growth rates and fermentation yields varied greatly. With the aim of optimizing growth on xylulose, we subjected these twelve strains to microevolution by serial dilutions on media containing xylulose as the sole carbon source (Fig. 1a). This process was carried out for a total of ~300 generations.

Measuring the growth curves of the evolved strains revealed that the strains underwent different types of growth improvements (Fig. 1b). For example, while some strains improved growth rate during logarithmic growth, others improved the saturation density, improving the overall yield. In attempt to describe the effect of evolution using a single metric, we first considered separately the logarithmic growth rate and the final OD level reached in saturation (Table 1). However, the evolutionary effect with respect to these two parameters was not consistent across all strains and we therefore examined for a different metric.

**Table 1:** Changes in growth parameters following evolution on xylulose

Strain	Fold change (evolved / wt)			
	Growth rate	Maximum OD	Time to maximum OD	Effective growth rate^a^
1	1.32	3	1.53	1.96
2	1.03	1.39	1.2	1.16
3	1.78	3.27	1.36	2.4
4	1.13	1.36	0.82	1.66
5	1.38	1.8	0.76	2.37
6	0.78	1.15	0.79	1.46
7	1.11	1.07	0.73	1.47
8	1.36	1.41	0.8	1.76
9	1.14	1.29	1.21	1.07
10	1.4	1.14	1.19	0.96
11	1.14	1.38	0.94	1.47
12	0.92	0.97	0.88	1.1

^a^Maximum OD / Time to maximum OD

To capture the different aspects of growth improvement using a single parameter, we defined an effective growth rate as the maximal OD level reached during growth divided by the time elapsed until saturation (following inoculation in xylulose media). By this measure, eleven of the twelve strains exhibited improved growth, while one maintained the same effective growth rate as its parent strain (Fig. 1c and Table 1).

### Evolved strains eliminated the AA-biosynthesis gene induction upon shift from glucose to xylulose

As described above, we previously noted that growth on xylulose induces expression of AA-biosynthesis genes. We asked whether this induction is maintained also in our evolutionarily-optimized strains. Using RNA-Seq, we characterized the transcription profiles of all strains in our collection, before and after evolution, during logarithmic growth on xylulose and on glucose. This was performed in two biological repeats, totaling ninety six genome-wide expression measurements. For each strain (before and after evolution), we defined the (log) ratio of gene expression in xylulose vs. glucose. We then systematically defined the groups of genes whose level of expression significantly changed upon shift from glucose to xylulose. This was done by first identifying all the genes whose level of expression changed significantly and then examining for functional enrichments by projecting this set of varying genes on pre-defined functional gene groups [22]. Thus, groups of genes were identified which were either induced or reduced in expression on xylulose compared to glucose, in pre-evolved wild type strains and in the evolved populations.

**Fig. 2:**
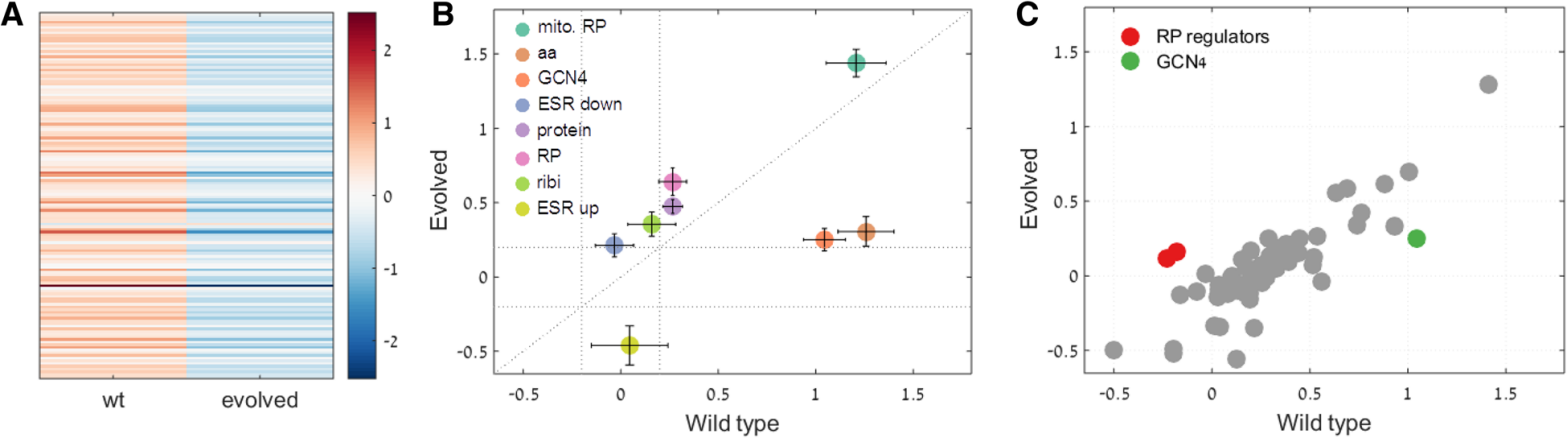
Gene group expression comparison. **a** Ratio between (log) expression on xylulose and glucose of all genes comprising the amino-acid biosynthesis expression module (*n* = 136), for wild type and evolved strains. Expression was averaged over all twelve strains and normalized per gene (materials and methods). **b** Wild type vs. evolved mean (log) expression (xylulose/glucose) of various gene groups, averaged over the twelve strains. Error bars represent standard error. Gene group names: mitochondrial ribosomal proteins (mito. RP), amino-acid biosynthesis (aa), GCN4-regulated (GCN4), genes down-regulated as part of the environmental stress response (ESR down), protein synthesis (protein), ribosomal proteins (RP), ribosome biogenesis and assembly (ribi) and genes up-regulated as part of the environmental stress response (ESR up). **c** Same as in B but for gene groups regulated by various transcription factors (*n* = 66, *gray dots*), RP regulators FHL1 and SFP1 (*red dots*) and the amino-acid biosynthesis regulator GCN4 (*green dot*)

Consistent with our prediction, the most significant difference in the pattern of induced expression before and after evolution was that of the AA-biosynthesis gene group (Fig. 2a, b). These genes were significantly induced in the ancestral strains (prior to evolution), but this induction was practically absent following evolution. This result was independent of how the group was pre-defined, either as a co-regulated module or by the targets of GCN4, the AA-biosynthesis master regulator (Additional file 1). The observed change in expression pattern was significantly larger than the inter-strain variation and was observed exclusively during growth on xylulose, but not on glucose (Additional file 2).

We further characterized the expression changes in gene groups associated with ribosome production (RP and Ribi), protein synthesis and the environmental stress response (ESR). Expression of these gene groups typically correlates with cellular growth rate. Expression of RP, Ribi and protein synthesis genes was up-regulated in xylulose compared to glucose following evolution (Fig. 2b). In addition, genes induced as part of the known environmental stress response [6, 23] were down-regulated in the evolved populations, while genes which are normally reduced in expression as a response to environmental stress were up-regulated. These results are consistent with the fact that the expression of these gene groups is correlated with growth rate and demonstrate adaptation at the level of gene expression. Here too, the observed effect resulted from changes in expression during growth on xylulose, rather than on glucose (Additional file 2).

Interestingly, in evolved strains, genes whose expression is typically induced in the presence of environmental stress were down-regulated on xylulose, compared to glucose (Fig. 2b). This down-regulation resulted from both reduced expression of these genes on xylulose and also increased expression during growth on glucose (Additional file 2). This may indicate a tradeoff in the efficiency by which glucose and xylulose are utilized, whereby adaptation to growth on xylulose was acquired, to some extent, at the expense of growth on glucose.

To determine whether the above changes in gene expression profiles are reflected in expression of regulatory elements, we examined for expression changes among a large set of gene groups characterized by the transcription factors associated with each group (*n* = 69). Notably, the most significant changes in expression on xylulose following evolution were the down-regulation of GCN4 and up-regulation of FHL1 and SFP1 (Fig. 2c). GCN4 is the master regulator of amino acid biosynthesis genes and its down-regulation following evolution is coherent with reduction in expression of this group of genes. Both FHL1 and SFP1 are known to be involved in the regulation of RP gene expression. SFP1 is also associated with Ribi gene expression regulation.

### The reduction in AA-biosynthesis gene expression correlates with effective growth rate improvement

**Fig. 3:**
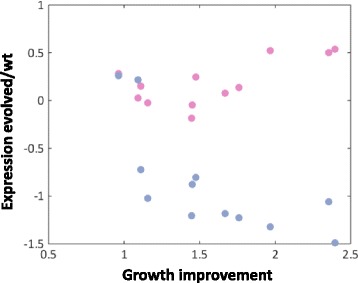
Correlation between expression and growth change. Ratio between effective growth rate on xylulose of each evolved strain and its corresponding wild type strain vs. (log) expression ratio between evolved and wild type strains for GCN4-regulated genes (*light blue*) and RP genes (*pink*)

The expression changes in the aforementioned gene groups as a result of evolutionary adaptation to growth on xylulose suggest that the extent of the change might be proportional to the degree of adaptation. To test whether this is the case, we examined the correlation, or lack thereof, of the expression changes of these genes groups with the degree of improvement in growth on xylulose, as defined by the ratio between effective growth rates after and before evolution. We found that the change in expression level (evolved/non-evolved) of the amino acid biosynthesis regulator – GCN4 – was anti-correlated with growth improvement (pv = 0.006, Fig. 3). In addition, the expression changes of ribosomal protein genes were (positively) correlated with growth improvement on xylulose (pv = 0.017). This was independent on how the group was defined; thus expression level change in amino acid biosynthesis genes was significantly anti-correlated with the level of growth improvement on xylulose (pv =0.008) while that of protein synthesis genes was positively correlated (pv = 0.009, Additional file 3). No correlation was observed between growth improvement and expression changes of other gene groups (Additional file 3).

## Discussion and conclusions

**Fig. 4:**
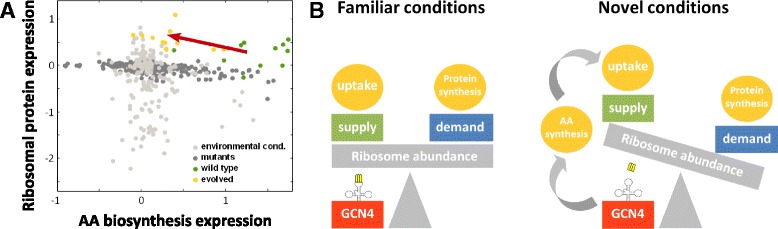
Balancing amino-acid supply and demand in familiar and novel conditions. **a** Expression of amino-acid biosynthesis genes vs. RP genes for various environmental conditions including temperature and osmotic shock, amino-acid starvation, nitrogen depletion, addition of hydrogen-peroxide/menadione/DTT (*light gray*, Gasch et al.), a set of gene deletion mutant strains (Hughes et al., *dark gray*), the twelve wild type strains used in this work (*green*) and the twelve evolved strains (*yellow*). *Red* arrow indicates the vector of change during evolution. **b** Model for amino-acid supply and demand balance. In a familiar environment, amino-acid supply and demand are balanced by evolutionarily tuned adjustment of ribosome abundance. In novel conditions un-optimized by evolution, a shortage in amino-acid results in accumulation of uncharged tRNAs, which acts as an internal signal for GCN4-mediated induction of amino-acid biosynthesis genes

We previously reported that cells growing on xylulose show a distinct expression pattern characterized by an induction of AA-biosynthesis group in a manner that was inversely correlated with the growth rate of the cell. This induction occurred despite the fact that the media was supplemented with all required amino-acids. Searching through a large dataset [22], we detected a similar general induction of this group of genes in a group of mutant strains deleted of a variety of genes [21], most of which are not related to AA metabolism (Fig. 4a). We reasoned that the two situations are related by the fact that both represent conditions that are not evolutionarily optimized: xylulose is not naturally available outside cells, so budding yeast were unlikely to frequently encounter it during the course of evolution. Similarly, deleting any particular gene modifies the internal state of the cell in a way that it did not undergo evolutionary optimization for. This indicated that induction of the AA-biosynthesis gene group may be a consequence of such non-optimized internal state.

In the preset study we examine this hypothesis by tested whether this induction is lost once the cells have had a chance to (evolutionarily) adapt to this new condition. Indeed, not only was the induction lost upon such adaptation, resulting in reduced expression of this group specifically in xylulose (Fig. 4a), but the improvement in effective growth rate was correlated with the extent to which AA-biosynthesis expression was reduced. While these results do not demonstrate a direct causal relationship between evolutionary improvement and the observed changes in gene expression, especially considering that at least two strains display similar improvement but different expression pattern, they strongly suggest that amino-acids biosynthesis forms a major bottleneck for growth in conditions not subject to evolutionary adaptation, as we postulated.

In order to achieve a balanced cellular level of amino acids, the incoming flux of amino acids must match the rate by witch proteins are synthesized. AA-biosynthesis is induced by accumulation of uncharged tRNAs, reflecting a demand for amino-acids that is larger than their supply. By contrast, expression of ribosomal-associated genes is largely dependent on signaling pathways such as TOR and PKA. This implies that in order to maintain balance between supply and demand, a tuning mechanism between these pathways is required, which we postulate is achieved primarily through evolutionary adaptation.

We propose a model comprised of two possible scenarios relating to this process (Fig. 4b). In an evolutionary ‘familiar’ environment, evolution had optimized signaling pathways to adjust the level of translation machinery with the expected rate of amino acid supply. However, in the absence of such evolutionary tuning, as may be exemplified by growth on xylulose or in the presence of genetic mutations, a transient imbalance occurs which triggers an internal correction mechanism activated through the induction of the GCN4-module. Subsequent evolutionary adaptation to the ‘unfamiliar’ environment will improve the tuning mechanism, thereby reducing GCN4-dependent gene expression.

The need for internal adjustment compromises cellular growth. Thus, according to this model and confirmed by our results, the first step in evolutionary adaptation to ‘unfamiliar’ conditions would be to optimize the balance between supply and demand of amino acids by adjusting the level of translation machinery, thereby abrogating the need for the internal ‘backup’ control of amino-acid biosynthesis adjustments.

Biotechnological applications often rely on engineering cells to either utilize new substrates or produce novel products. For example, a major limitation of using budding yeast as an economically viable solution for generating biofuel is its inability to ferment xylose, motivating engineering efforts for establishing a xylose-utilizing budding yeast strain. These engineered strains may experience a similar situation to the one we describe above, in that they are put in a condition not optimized by evolution. Our results suggest that co-optimizing amino-acid metabolism may be useful for improving growth performance of these strains.

## Methods

### Strains and media

A complete description of wild type yeast strains used in this study can be found in [17]. YP-xylulose medium was prepared as described in [24]. Briefly, D-xylose was enzymatically converted to D-xylulose using xylose isomerase (Sigma-Aldrich). This yielded a 70:30 mixture of xylose:xylulose, as measured by HPLC (Agilent 1200 series). YP media was supplemented with the pentose mixture to a final concentration of 2 g/L xylulose. Although growth on xylose was previously reported for certain wild type *S. cerevisiae* strains [25], in our experiments xylose was not utilized by any of the strains during the growth period, as determined by HPLC analysis of the growth media after reaching saturation.

#### Evolution and growth measurements

All laboratory evolution experiments were carried out by serial dilution in 24-well plates with breathable seals. Plates were grown on an orbital shaker at 600 rpm in 30 °C. Each evolving strain was grown on 1.2 ml of YP-xylulose medium until reaching stationary phase and then diluted daily by a factor of 1:64 into fresh media (6 generations per dilution). Growth of wild type and evolved populations was characterized using a Hamilton robotic system. Cultures were grown in 96-well plates with 600 rpm shaking in 30 °C. Mineral oil was added to each well to prevent evaporation. OD_600_ measurements were taken during growth using a Tecan F500 plate reader.

#### RNA sequencing and normalization

Total mRNA of each of the twelve strains from our wild type yeast strain collection, both before and after evolution, was sequenced, during growth on glucose and during growth on xylulose. Two repeats were performed for each strain and condition, thus totaling 96 expression measurements. Prior to RNA extraction, populations of each strain before and after evolution were grown on YP media supplemented with either glucose or xylulose. Cells were harvested at mid log phase and frozen immediately in liquid nitrogen. For total RNA extraction, cell lysis was performed in a 96 deep-well plate by adding 450 μl of lysis buffer containing 1 M sorbitol (Sigma-Aldrich), 100 mM EDTA and 0.45 μl lyticase (10 IU/μl). The plate was then incubated in 30 °C for 30′ and centrifuged for 10′ at 2500 rpm after which the supernatant was removed. RNA extraction proceeded by following the NucleoSpin® 96 RNA kit (Macherey-Nagel) protocol, substituting β-mercaptoethanol with DTT. RNA extracts were subsequently fragmented and selected for polyadenylation using PolydT beads. Barcoded cDNA was prepared using Smartscribe and unique molecular identifier (UMI)-containing primers [26], and subjected to RNase H treatment. A second adaptor was ligated, and cDNA was amplified by PCR using KAPA HiFi DNA polymerase for 15 cycles. cDNA was sequenced using an Illumina HiSeq2000 platform with 50 bps single reads. Reads were mapped to the *S. cerevisiae* S288c reference genome (SGD R64) using Bowtie. Reads mapping to rRNA were filtered out. Normalization for PCR bias was carried out using UMI scoring [26]. The total number of mapped reads per sample was normalized independently to 106.

The expression value of each gene in each strain, either before or after evolution, was averaged over two repeats. Except for the individual amino-acid genes expression comparison (Fig. 2a), all expression values of gene groups before and after evolution were obtained by averaging the number of mapped reads per gene in the gene group, per strain, and then averaging across all strains either before or after evolution. For comparison of amino-acid biosynthesis genes expression between populations before and after evolution (Fig. 2a), the mean expression level of each gene before and after evolution was first calculated by averaging over all strains and the two mean values were normalized by the total number of reads per gene across all samples.

## Additional files

Additional file 1: GCN4-regulated genes expression comparison. (PDF 175 kb)

Additional file 2: Gene group expression comparison between sugars. (PDF 188 kb)

Additional file 3: Correlation between expression change of various gene groups and growth change. (PDF 299 kb)

## Abbreviations

ESR: Environmental stress response
Ribi: Ribosome biogenesis
RP: Ribosomal proteins
UMI: Unique molecular identifier
