# Supplementary material for: Rapid evolutionary adaptation to growth on an ‘unfamiliar’ carbon source
Source: BMC Genomics. 2016 Aug 24;17:674. doi: 10.1186/s12864-016-3010-x (PMC5477773; doi:10.1186/s12864-016-3010-x)
Supplement: Supplementary file 1 — GCN4-regulated genes expression comparison. (PDF 175 kb) [file 12864_2016_3010_MOESM1_ESM.pdf]

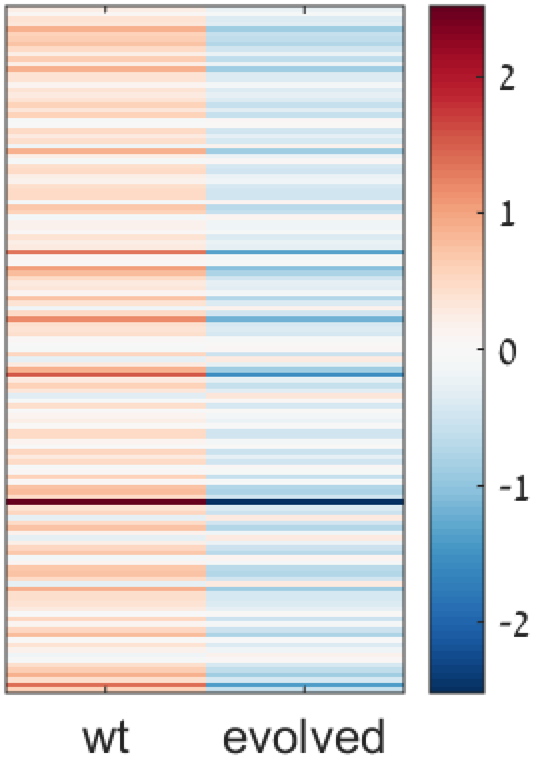

GCN4-regulated genes expression comparison. Ratio between (log) expression on xylulose and glucose of all GCN4-regulated genes for wild type and evolved strains. Expression was averaged over all twelve strains and normalized per gene (materials and methods).
