# Supplementary material for: Rapid evolutionary adaptation to growth on an ‘unfamiliar’ carbon source
Source: BMC Genomics. 2016 Aug 24;17:674. doi: 10.1186/s12864-016-3010-x (PMC5477773; doi:10.1186/s12864-016-3010-x)
Supplement: Supplementary file 2 — Gene group expression comparison between sugars. (PDF 188 kb) [file 12864_2016_3010_MOESM2_ESM.pdf]

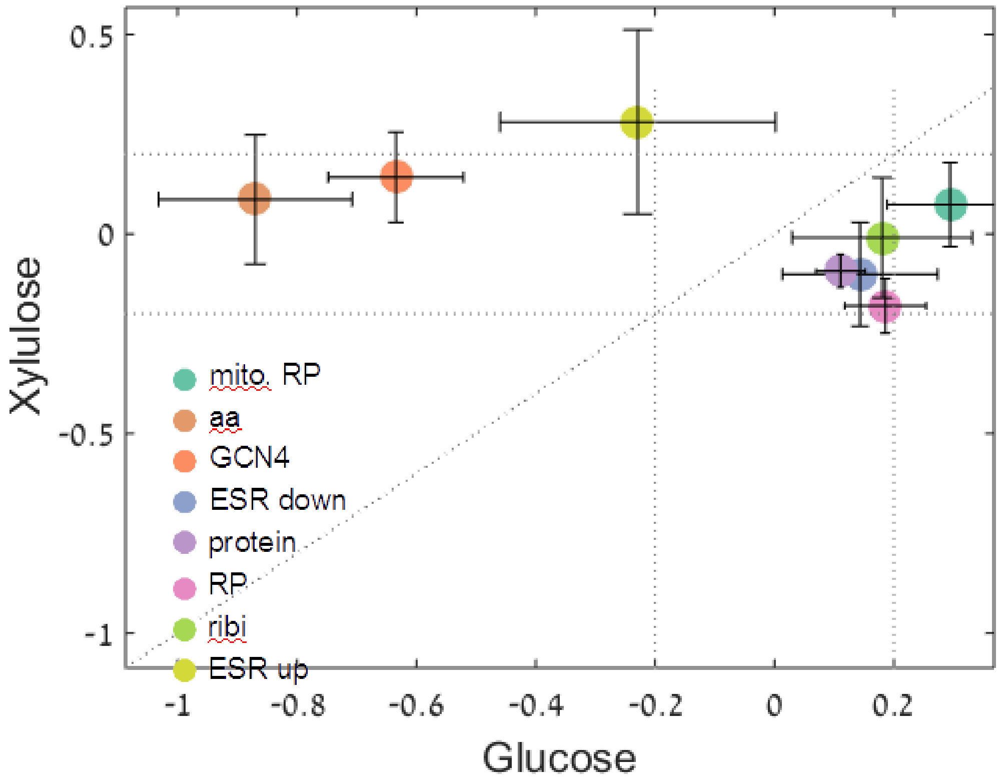

Gene group expression comparison between sugars. Mean (log) expression ratio (evolved/wild-type) on xylulose vs. glucose of various gene groups, averaged over the twelve strains. Gene group names: mitochondrial ribosomal proteins (mito. RP), amino-acid biosynthesis (aa), GCN4-regulated (GCN4), genes down-regulated as part of the environmental stress response (ESR down), protein synthesis (protein), ribosomal proteins (RP), ribosome biogenesis and assembly (ribi) and genes up-regulated as part of the environmental stress response (ESR up).
