# Supplementary material for: Rapid evolutionary adaptation to growth on an ‘unfamiliar’ carbon source
Source: BMC Genomics. 2016 Aug 24;17:674. doi: 10.1186/s12864-016-3010-x (PMC5477773; doi:10.1186/s12864-016-3010-x)
Supplement: Supplementary file 3 — Correlation between expression change of various gene groups and growth change. (PDF 299 kb) [file 12864_2016_3010_MOESM3_ESM.pdf]

A

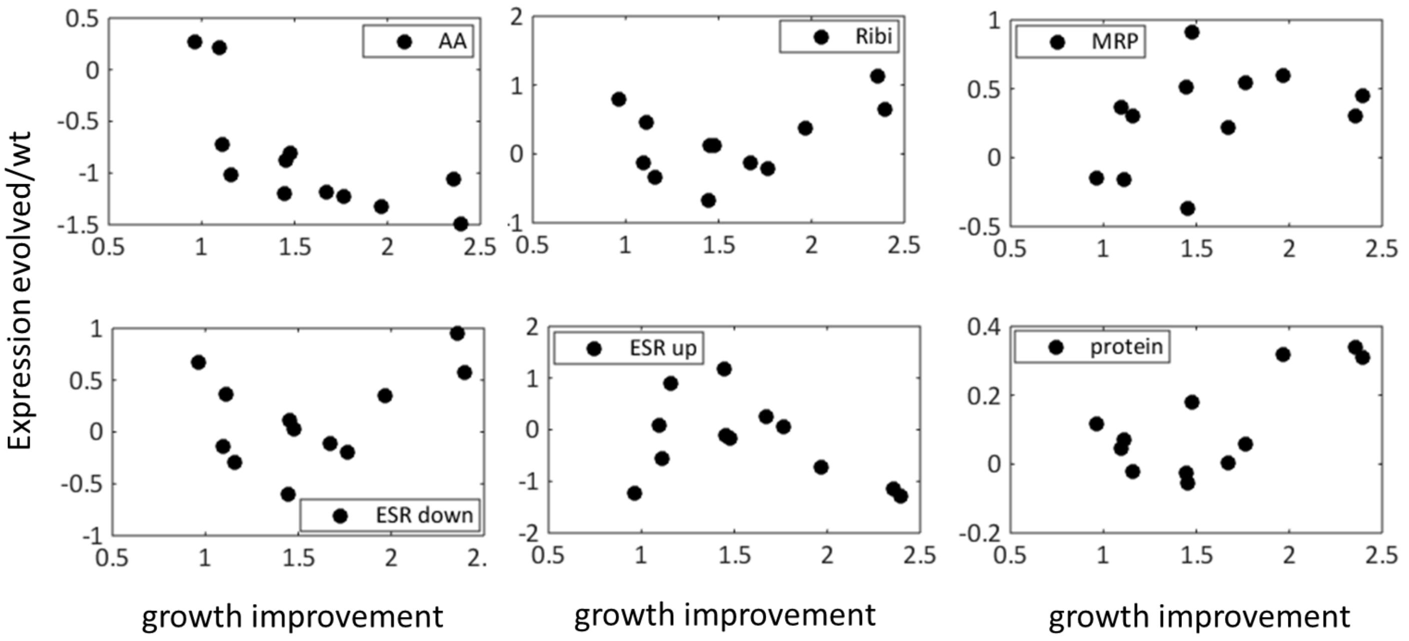

B

	AA	Ribi	MRP	ESR down	ESR up	Protein synthesis
**Correlation coefficient**	-0.72	0.4	0.38	0.41	-0.41	0.72
**PV**	0.008	0.2	0.22	0.18	0.18	0.009

Correlation between expression change of various gene groups and growth change A) Ratio between effective growth rate on xylulose of each evolved strain and its corresponding wild type strain vs. (log) expression ratio between evolved and wild type strains for various gene groups including amino acid biosynthesis (AA), Ribosome biogenesis and assembly (Ribi), Mitochondrial ribosomal proteins (MRP), genes down-regulated as part of the environmental stress response (ESR down), genes up-regulated as part of the environmental stress response (ESR up) and protein synthesis genes. B) Pearson correlation coefficient and corresponding p-value for the significance of the correlation between growth improvement as defined in A and each gene group in A.
